# Supplementary material for: Methane emissions from US low production oil and natural gas well sites
Source: Nat Commun. 2022 Apr 19;13:2085. doi: 10.1038/s41467-022-29709-3 (PMC9019036; doi:10.1038/s41467-022-29709-3)
Supplement: Supplementary file 1 — Supplementary Information [file 41467_2022_29709_MOESM1_ESM.pdf]

Supplementary Note 1: Aggregation of O&G well-level dataset

We use the monthly O&G well-level and production data available from Enverus Prism[1], a commercial platform which collects and aggregates public and proprietary O&G data for wells in the US For the year 2019, we aggregated the production data to annual production metrics for 2019 based on each well's unique well ID. The summary statistics for this initial data aggregation are shown in Supplementary Table 1 below.

Supplementary Table 1: Summary statistics for data aggregation of Enverus Prism dataset for 2019.

	Total number of wells	Gross oil production (billion barrels)	Gross gas production (trillion cubic feet, Tcf)	O&G production (billion boe)	Avg # of production days per year per well
Raw data (2019)	867,204	4.4	41	11	325
Filtered O&G onshore wells	842,978	3.7	39	10	325

Ninety four percent of all the wells in the database had data on the operator-reported total number of production days. Among the wells with non-zero production days in 2019, 666,712 wells reported total production days of over 300 days in 2019 (Supplementary Fig. 1). The average number of reported production days per well in 2019 was 325 days (Supplementary Table 1).

Supplementary Fig. 1. Histogram of reported number of production days per well in 2019. Data from Enverus Prism[1]

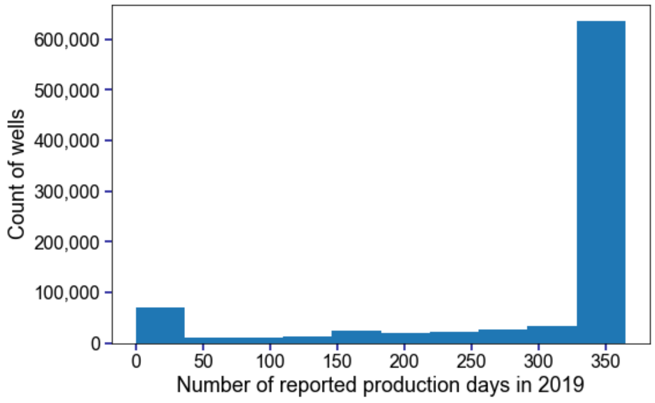

Given the available information on the number of production days per well, we calculated each well's average combined oil and gas production in units of barrels of oil equivalent per day (boed) as follows (Supplementary Eq. 1), where d is the reported number of production days for each well, and assuming one barrel of oil equivalent (boe) has the energy equivalent of 6,000 cubic feet of gas or 6 Mcf[2]. For the four percent of wells with no operator-reported data on the number of production days, we assumed 365 days of production in 2019.

$$\frac{boed}{well} = \frac{1}{d}(OIL\ [barrels] + \frac{GAS[Mcf]}{6}) \quad \text{[Supplementary Eq. 1]}$$

Next, we filtered the aggregated dataset for specific well types for which we did not have methan e emissions data. These are based on Enverus Prism's-derived well types and included cola-bed methane wells, water wells, brine wells, CO2 injection wells, and service, storage and disposal w ells.

This resulted into a total of 849,611 wells. We then filtered the dataset for wells located on only onshore locations, using the shapefile data for US states available from the US Census Bureau[3]. As part of this assessment, we excluded wells that were identified in Enverus Prism to be in offshore counties or basins while located within the state boundaries.

The summary statistics for the final dataset are shown in Supplementary Table 1.

Supplementary Note 2: Clustering well-level data into well-site data

Because CH_4 emissions data are reported at the site-level, and given that each site can have one or multiple wellheads on the site, we clustered the well-level data to generate site-level data and related attributes. The geospatial analysis procedure used here builds upon previous methods[4,5] by separately assessing each well based on its location and drill trajectory. The location information allows us to define the appropriate projected coordinate reference system for the well. In addition, a review of the locations of a select fraction of wells in satellite imagery indicated that the older, vertically-drilled wells are generally smaller in size compared to the newer, horizontally-drilled wells. Furthermore, the majority of low production wells (85%) are vertically-drilled, while most of the non-low production wells are horizontally-drilled (60%; Supplementary Fig. 2).

Supplementary Fig. 2. Low production wells by drilling trajectory.

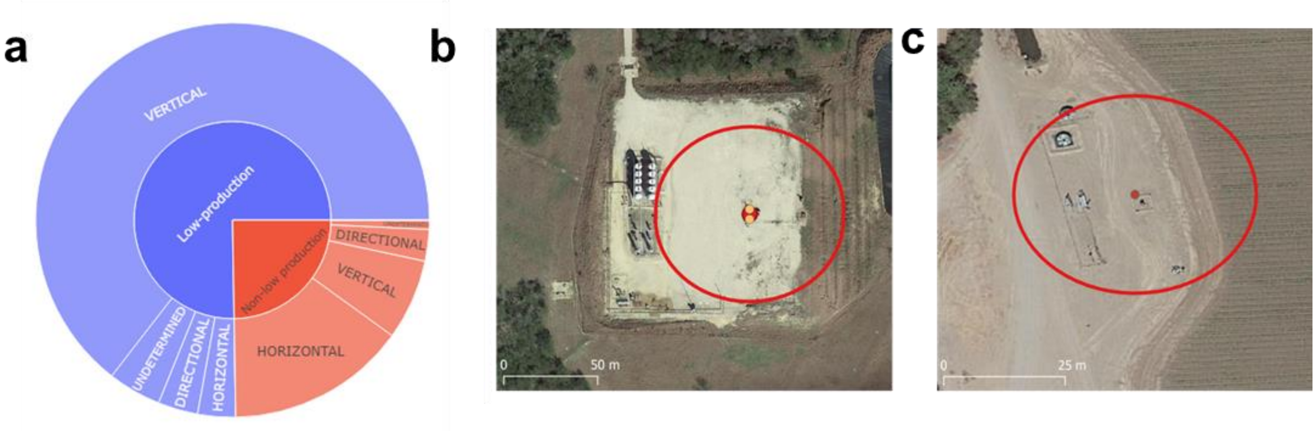

a Distribution of US O&G wells by low production/non-low production category and by drilling trajectory (vertical, directional, horizontal, and undetermined); **b** An example of a satellite image of a low production well site with two horizontally-drilled wells; **c** An example of a satellite image of a low production well site with a single vertically-drilled well. In all cases, the red circle represents the 50-m or 25-m radius buffer around each well location. Basemap imagery from Google Maps. Data based on authors' analysis of Enverus Prism data.

The clustering of spatial well-level data to site-level information requires an appropriate choice of buffer radius. To test for sensitivity to buffer radii, we selected 10,000—20,000 wells in each of the top 15 US states (OK, PA, NY, WV, OH, KS, NM, CO, TX, CA, WY, LA, AR, KY, and ND) for O&G production and performed geospatial clustering analysis on the well-level locations at various buffer radii ranging from 10 m to 100 m, treating vertically- and directionally-drilled wells (including those with unknown drilling trajectory) separately from horizontally-drilled wells. As buffer radius is increased, more wells are aggregated to form well sites, resulting into fewer well sites. An optimal buffer radius can be assessed based on the successive percentage changes in the number of well sites as buffer radii is increased: at optimal buffer radius, a minimum percentage change, or a leveling off in the percentage change in the number of well sites, is observed (Supplementary Fig. 3).

Supplementary Fig. 3. Sensitivity to buffer radius for geospatial clustering of spatial data for wells.

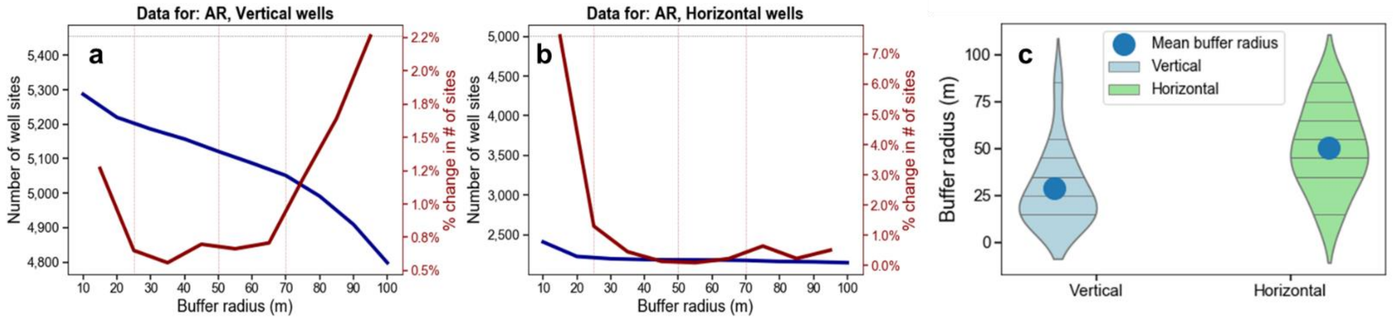

a An example of geospatial clustering of wells in Arkansas; vertical wells include vertical, directional, and deviated wells or wells of undetermined/unknown trajectory. **b** An example of geospatial clustering of wells in Arkansas for horizontally-drilled wells. In both a and b, the right y-axis (solid dark red line) shows the successive percent change in the number of well sites (e.g., between 10-m and 20-m radii and between 20-m and 30-m radii choices). The minimum percent change is assumed to occur at an optimal buffer radius for well sites in the state. **c** Distribution of optimal buffer radii for the top 15 US states, indicating a mean of ~25-m and 50-m buffer radius for vertically-drilled and horizontally -drilled wells, respectively.

Supplementary Fig. 3 shows the distribution of the optimal buffer radius for vertically-drilled wells and horizontally-drilled wells. Based on this sensitivity analysis, confirmed by visual, qualitative assessment of a select number of well sites in satellite imagery, a buffer radius of 25 m and 50 m was selected for geospatial clustering of vertically-/directionally-drilled and horizontally-drilled well sites, respectively. For each well with known location (lat/lon), we applied an appropriate coordinate reference system based on the European Petroleum Survey Group's (EPSG) codes for the specific UTM Zone for each location. A 25-m or 50-m buffer was then generated around each well location, depending on well drilling trajectory, and overlapping buffers were spatially merged. For multi-well sites, each well's production attributes were summed to yield site-level attributes. Supplementary Fig. 4 shows examples of single-well and multi-well sites for low production well sites as seen in satellite imagery (imagery basemap: Google Maps).

Using this approach, the total number of active onshore well sites, defined as a well site with total combined O&G production > 0 boed/site, was 700,000 (3 significant figures) well sites in 2019, representing an average of 1.2 wells per site. The distribution of the number of wells per site is shown in Supplementary Fig. 5.

Supplementary Fig. 4. Examples of low production well sites as seen in satellite imagery.

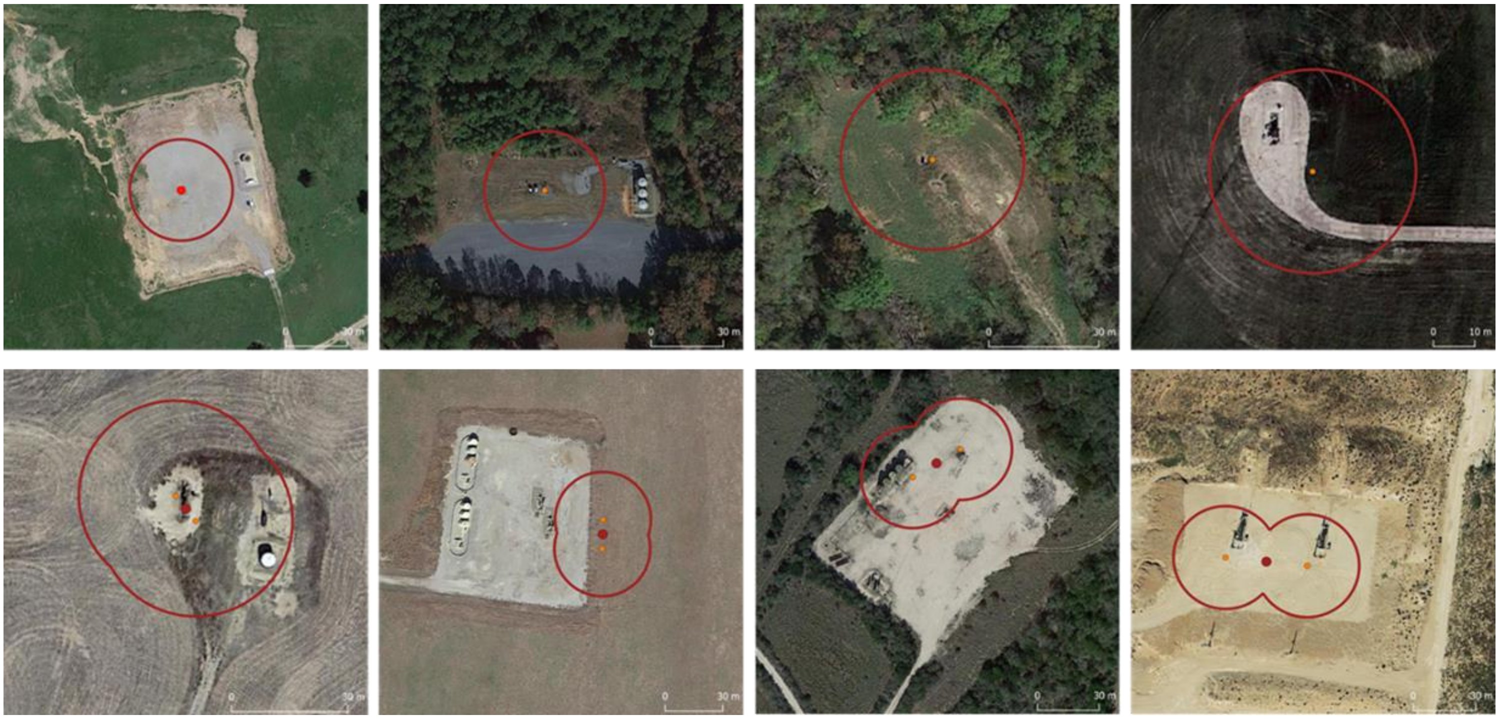

The top row shows single-well sites; the bottom row shows multi-well sites. In all cases, the red circles represent a 25-m radius buffer around each wellhead's location. For multi-well sites, the overlapping buffers are merged and site-level O&G production obtained by summing the production data for all the wells on the site. Imagery basemap from Google Maps.

Supplementary Fig. 5. Distribution of the number of wells per site.

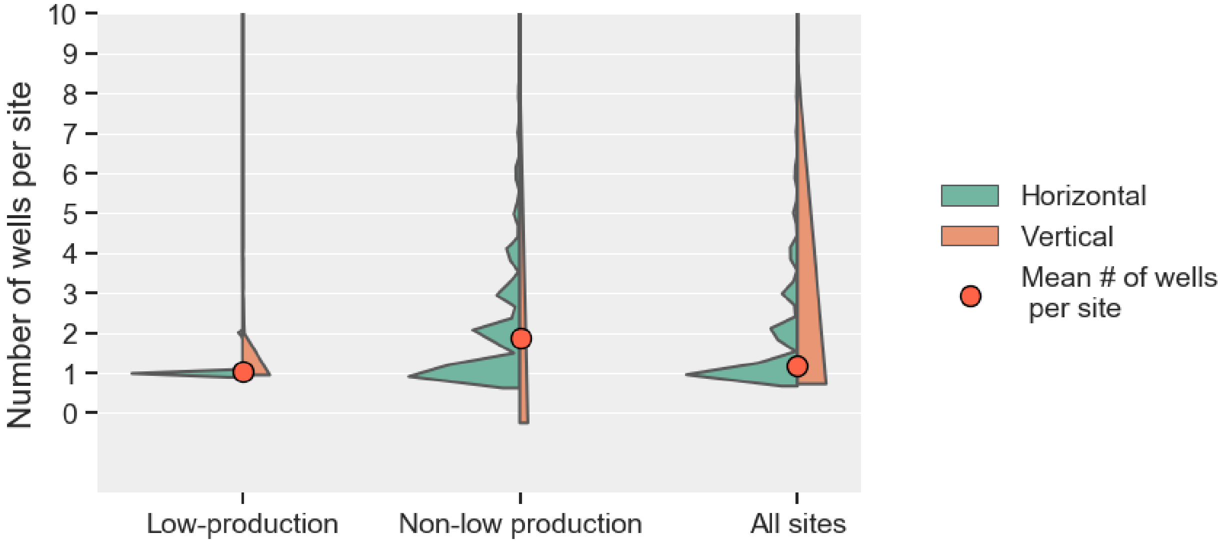

Violin plots showing the distribution of number of wells per site for low production well sites, non-low production well sites, and all US O&G well sites. For visualization, we show the distribution for sites with up to 10 wellheads each (see discussion below regarding uncertainty assessment). For each production category, we distinguish between vertical wells (includes wells of vertical, directional, deviated and unknown drill trajectory) and horizontal wells (includes horizontally-drilled wells). All data are based on an analysis of Enverus Prism well-level data for 2019.

There are uncertainties in the estimates for the total number of well sites and their production that are difficult to quantify based on available data. These include (i) uncertainty in location data for wells and (ii) inaccuracies in the operator-reported data. In addition, some states, e.g., Texas, require operators to report production data at the lease-level, not at the well-level. Data from Enverus Prism are allocated to wells on each lease using proprietary algorithms with unknown precision and accuracy. We use the data as is (accessed in June 2021 from Enverus Prism platform). Notwithstanding uncertainties associated with location inaccuracies or reporting errors, we estimate an overall error in the estimated total number of well sites of +2%/-5% based on a sensitivity analysis where the buffer radius is varied from 10 m to 50 m for vertically-drilled wells and 20 m to 70 m for horizontally-drilled wells.

Supplementary Fig. 6. Contribution of low production well sites to the total O&G well site count, oil, gas and combined O&G production in 2019.

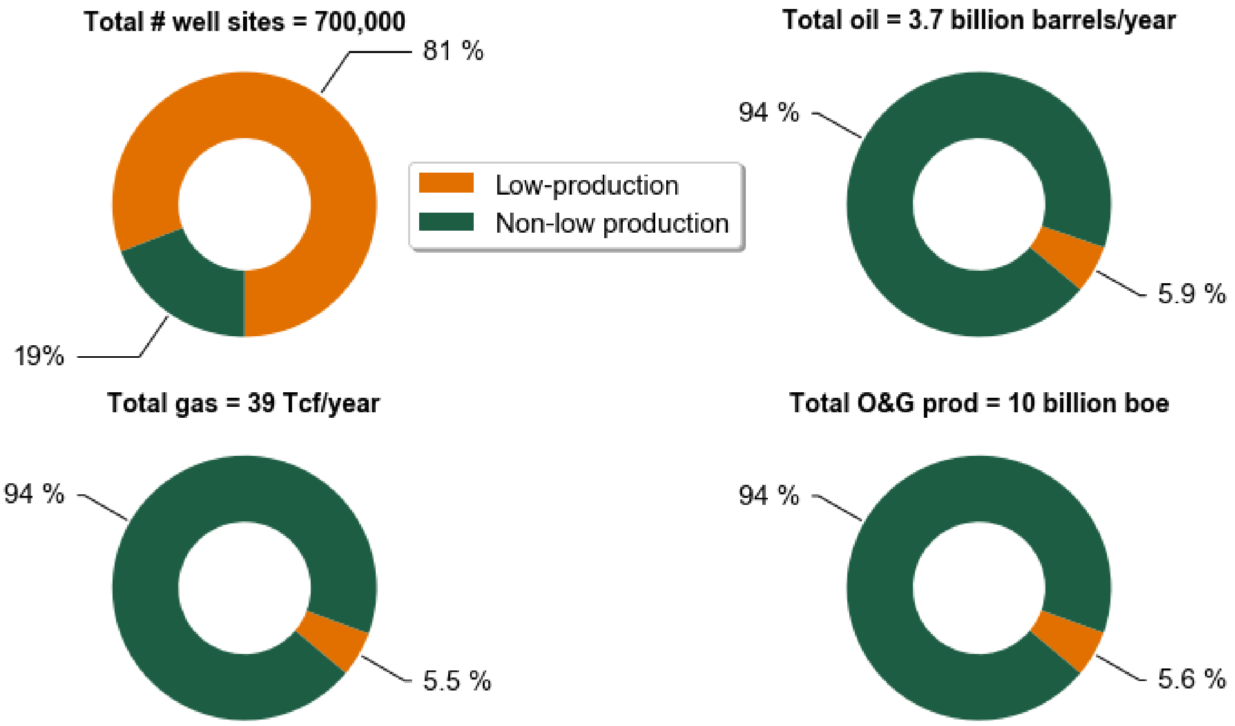

All data are based on an analysis of Enverus Prism well-level data for 2019.

Supplementary Fig. 7. US low production well site data

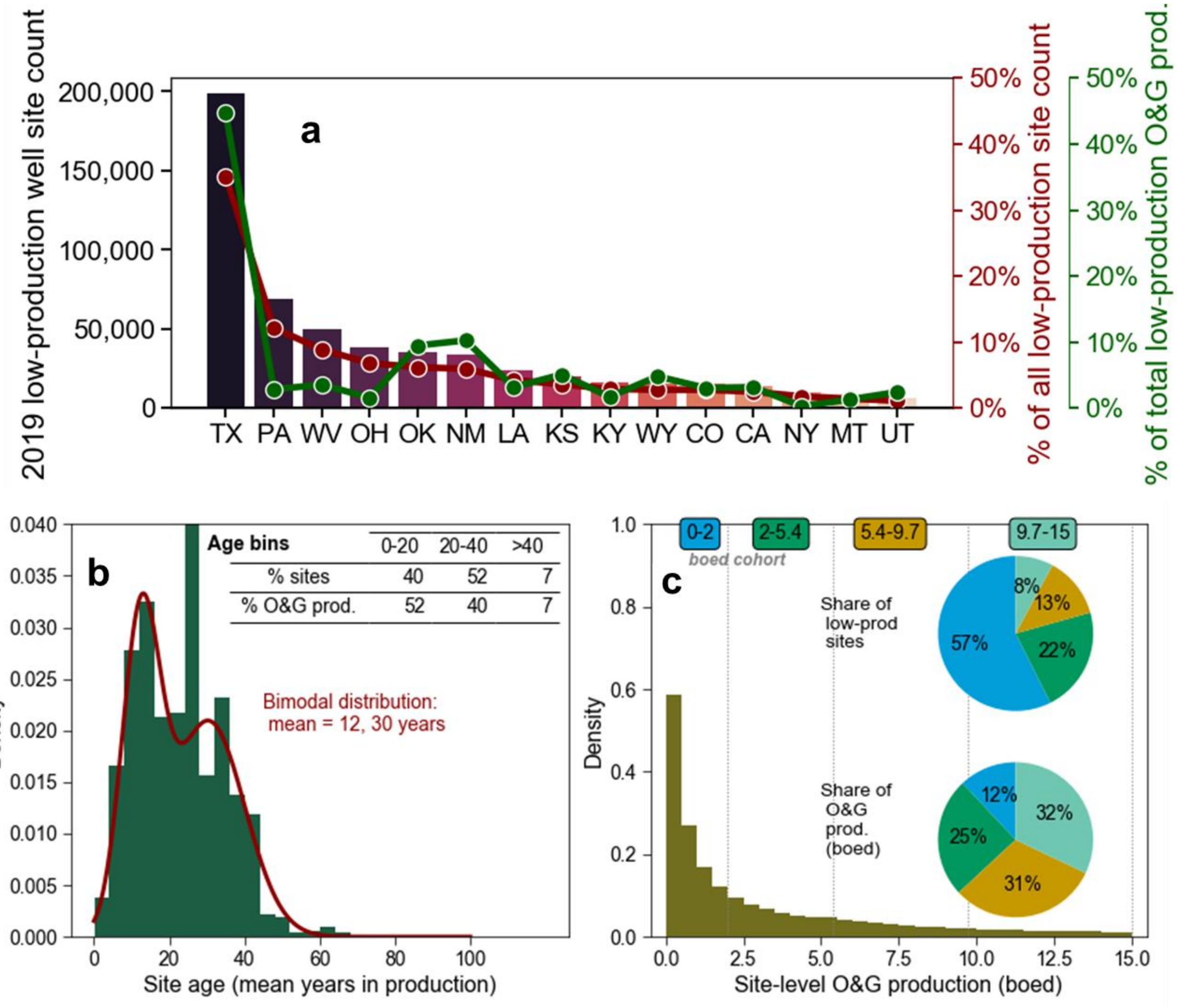

a Number of low production well sites for the major O&G producing states. The right y-axes show the percent of all low production well site count (dark red lines) and O&G production (green line) contributed by each state. **b** A histogram of mean well site age for low production well sites in the US The distribution is roughly bimodal, with peaks at ~12 years and 30 years. The inset table shows the percent of low production well sites and O&G production for each site age cohort of 0-20, 20-40 and >40 years. **c** A histogram of site-level O&G production for low production well sites, showing a large proportion of sites (57%) produce < 2 boed/site. The production cohort shown on the top x-axis is based on the natural breaks in the data obtained using the Jenks Natural Breaks algorithm, applied to all low production well site data. The inset pie charts show the share of low production well sites and O&G production by production cohort. All data are based on an analysis of Enverus Prism well-level data for 2019.

Supplementary Fig. 7 shows the distribution of low production well site count for the major US O&G producing states, indicating that Texas and the Appalachian states of Pennsylvania, West Virginia and Ohio dominate the total number of low production well sites nationally. Additionally, the distribution for site age indicates a bimodal distribution (Supplementary Fig. 7b), with peaks around 12 years old and 30 years old. Finally, as shown in Supplementary Fig. 7c, the majority of low production well sites (57%) produce <2 boed/site.

Supplementary Note 3: Production decline curves for low production well sites

In order to investigate the average time in which a newly completed well site reaches the threshold of low-productivity, we used Enverus Prism[1] data to track the reported production for all new wells that came online between January 2012 and October 2020. Supplementary Fig. 8 below shows the number of new wells in each month during this period.

Supplementary Fig. 8. Number of new wells that reported production for the first time in each month from January 2012 through October 2020.

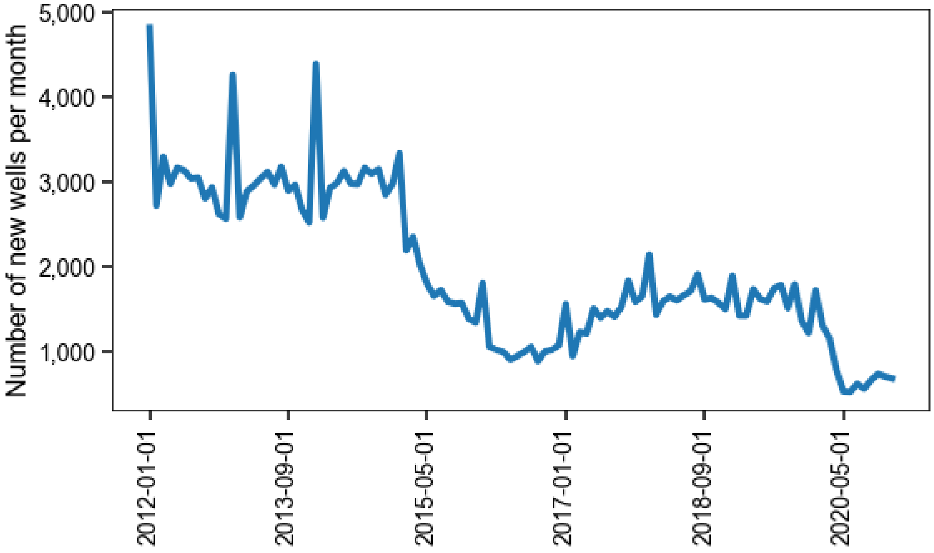

Analysis of data from Enverus Prism[1].

To simplify our analysis, we focused on those wells that we classified as single-well low production well sites that were operational in 2019, tracing their monthly production history forward from the first reported production date. This resulted into a total of 44,649 low production well sites that were actively producing in 2019 and had their first reported production date between 2012 and 2019.

If the first production date is assigned as month zero for each well, it is possible to track their monthly production in subsequent months and generate a production decline curve based on reported data. Grouping the data by month from first month of production to last reported month (in 2019), we compute the average boed for each month for all sites using Supplementary Eq. 1. Supplementary Fig. 9 shows the average production decline curve for the 44,649 single-well sites (84% vertically-drilled, 16% horizontally-drilled) that were low production sites in 2019 and had their first reported production date between 2012 and 2019. For these sites, site-level O&G production fell to below 15 boed/site generally within the first one to two years (for vertically-drilled wells) and two to five years (for horizontally-drilled wells) following initial production (Supplementary Fig. 9).

Supplementary Fig. 9. Production decline curve for 44,649 single-well low production well sites that were actively producing in 2019 and had their first reported production date between 2012 and 2019.

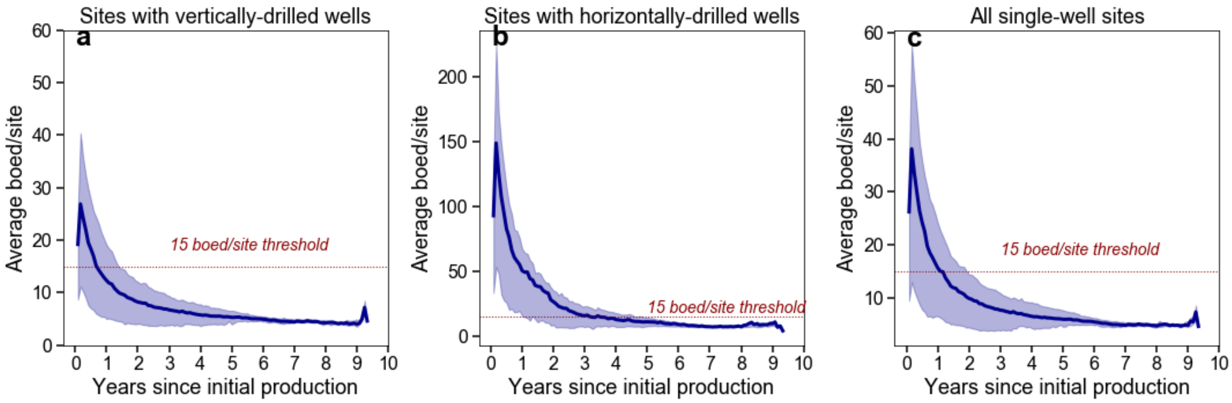

The solid blue line is the average O&G production rate (boed/site) at each month from first reported production date, while the shaded blue area represents the 25th and 75th percentiles. **a** Single-well sites with vertically-drilled wells, and includes directional and deviated wells. **b** Single-well sites with horizontally-drilled wells. **c** All single-well sites.

Supplementary Note 4: Review of site-level measurement-based data

We reviewed data from previously reported site-level measurement studies and selected study data based on the following criteria (see Main text):

(i) The measurements were focused on quantifying total site-level CH_4 emissions,
(ii) Measurements captured both low and high-emitting sites, and
(iii) Both oil and gas production data were reported for each site where they could be obtained (e.g., based on proprietary data, state-level reports or other reported attributes such as location of the measured site and date of measurement). We were not able to use data from site-level studies that did not report, or for which it was difficult to obtain, both oil and gas production data, as these production data were needed to assess the production category of sampled sites.

The summary statistics for O&G CH_4 emissions and site-level production as reported in selected previous studies are shown in Supplementary Table 2.

Supplementary Table 2. Summary statistics of previously published studies on well site CH_4 emissions

		size	Oil and gas production (boed)			Site-level CH_4 (kg/h)		
**study**	**basin**	**count**	**min**	**max**	**mean**	**min**	**max**	**mean**
**Brantley et al. (2014)**[6]	**Barnett**	13	0.6	14.7	9.4	0.1	3.5	1.2
	**Denver-Julesburg**	48	0.9	14.3	6.4	0.0	6.2	1.0
	**Eagle Ford**	1	13.0	13.0	13.0	4.8	4.8	4.8
	**Upper Green River**	18	1.5	12.9	6.4	0.1	28.4	3.8
**Caulton et al. (2019)**[9]	**Appalachian**	61	1.0	15.0	7.3	0.0	136.0	4.4
**EDF-PermianMap***	**Delaware**	10	0.2	8.4	1.7	0.0	0.6	0.1
**Omara et al. 2016**[4]	**Appalachian**	18	0.1	7.3	2.7	0.0	4.5	1.1
**Omara et al. 2018**[7]	**Denver-Julesburg**	8	0.2	14.6	5.5	0.1	9.3	3.2
	**Uinta**	9	0.7	13.9	6.6	0.3	6.5	3.1
**Robertson et al. (2017)**[8]	**Denver-Julesburg**	8	1.2	10.1	4.5	0.1	4.1	1.3
	**Fayetteville**	5	3.6	12.3	6.9	0.0	1.2	0.3
	**Uinta**	15	1.6	13.2	8.5	0.1	46.5	4.6
	**Upper Green River**	11	5.8	14.6	11.4	0.1	1.0	0.6
**Robertson et al. (2020)**[10]	**Delaware**	15	0.0	14.6	7.0	0.0	34.6	4.2

*The 10 site-level data for the Permian region comes from additional measurements conducted by the same team from University of Wyoming that published similar data for the Permian region based on previous OTM-33A measurements in 2018 (Robertson et al. 2020). This campaign was conducted as part of EDF's PermianMAP campaign (www.PermianMAP.org). The study design, methods and site-selection approaches are similar to Robertson et al. 2020's approach, and is fully described in Robertson et al. (2020).[10] The new data for the 10 low production well sites are included in Supplementary Data 1 and also publicly available at PermianMAP.org.

Supplementary Table 3 provides a summary of the selected previously reported measurement studies at O&G well pad sites that included relevant data on low production well site CH_4 emissions.

Supplementary Table 3. Summary of selected previously published studies on well site CH_4 emissions

	**Study summary**
Brantley et al. (2014)[6]	Brantley et al. performed OTM-33A methane emissions measurements at 228 O&G well pads in Texas (Barnett, Eagle Ford), Colorado (Denver-Julesburg), and Wyoming (Upper Green River). The study's method provides site-level quantification with a detection limit of 0.01 g/s (or 0.036 kg/h). A total of 80 sites were low-producing during the month of measurement, with combined O&G production per site ranging from 0.6 to 14.7 boed. For these sites, the reported site-level methane emissions ranged from 0.05 to 28 kg CH4/h. The study reported that *"Methane emissions were positively correlated with gas production, but only approximately 10% of the variation in emission rates was explained by variation in production levels. The weak correlation between emission and production rates may indicate that maintenance-related stochastic variables and design of production and control equipment are factors determining emissions."*<br><br>*Low production well site data used in this study?* **Yes**

Omara et al. (2016)[4]	Omara et al. used the tracer flux measurement approach to quantify site-level methane emissions at 18 low production well sites in SW Pennsylvania and N West Virginia. The sites had gas production ranging from 0.68 to 44 Mcfd and combined O&G production in the range of 0.12 to 7.8 boed. The reported site-level methane emissions ranged from 0.02 to 4.5 kg/h/site. This study also quantified methane emissions at 17 unconventional gas well pads in the Marcellus and found significant differences between the emissions from low production and non-low production well sites. The authors observed that *"these differences were attributed, in part, to the large size (based on number of wells and ancillary NG production equipment) and the significantly higher production rate of UNG sites. However, C_vNG sites generally had much higher production-normalized CH_4 emission rates (median: 11%; range: 0.35—91%) compared to UNG sites (median: 0.13%, range: 0.01—1.2%), likely resulting from a greater prevalence of avoidable process operating conditions (e.g., unresolved equipment maintenance issues)"*<br><br>*Low production well site data used in this study?* **Yes**

Robertson et al. (2017)[8]	Robertson et al. collected OTM-33A methane emissions data at 150 sites in the Upper Green River (Wyoming), the Denver-Julesburg (Colorado), Uinta Basin (Utah), and Fayetteville in 2014 and 2015. Measurements in the UGR, DJB and Uinta were performed on public roads while measurements in Fayetteville were performed either on public roads or on site access roads because the authors had site access granted by an anonymous operator. The authors reported that for well sites in the Fayetteville, Uinta and DJB, 20% of sites accounted for 72-83% of cumulative methane emissions, indicating the presence of high emitting sites. The authors also reported that average methane mass emission rates per well pad were similar among different basins despite large differences in average gas production, with the exception of Fayetteville which was dominated by dry gas production. The authors excluded any sites where an operator was visibly present and maintaining equipment, but reported that *"episodic events (e.g., flash emissions, automated liquid unloadings) and failed components (e.g., thief hatch stuck open, malfunctioning pressure relief valves) may have been captured during a measurement."* From this study, 39 sites were low production (i.e. <15 boed) and combined O&G production in the range of 1.2 to 14.6 boed. The measured methane emission rates for low production well sites ranged from 0.02 to 47 kg CH_4/h/site.<br><br>*Low production well site data used in this study?* **Yes**
Omara et al. (2018)[7]	This study included measurements at 92 O&G well pads in Uinta, Denver-Julesburg and Marcellus (NE PA) regions. Methane measurements were performed using a combination of measurement techniques, including the dual tracer flux, OTM-33A and mobile transects followed by Gaussian dispersion modeling (GDM). 17 sites measured in the Uinta and DJB were low production sites. Their combined O&G production ranged from 0.2 to 14.6 boed. The measured CH_4 emission rate ranged from 0.06 to 9.3 kg CH4/h.<br><br>*Low production well site data used in this study?* **Yes**

Caulton et al. (2019) [9]	Caulton et al. performed site-level methane emissions quantification at 677 unique well pads in Pennsylvania in 2016/2017. The study used mobile transects with Gaussian dispersion modeling techniques for quantifying methane emissions. They found that the distributions were "extremely" skewed, with the top 10% of emitters contributing 77% of total methane emissions. For all sites, the authors reported a methane loss rate of 0.53% with a 95% CI of 0.46 to 0.64%. We identified 61 sites that were low production sites in this dataset, filtering for only actively producing sites at the time of measurement. Using the reported site location, we also reviewed available data from Enverus Prism for the selected low production well sites to confirm that they were indeed low-producing at the time of measurement. Their reported gas production ranged from 6 to 90 Mcfd and combined O&G production ranged from 1 to 15 boed. The reported methane emissions ranged from 0 to 136 kg CH_4/h. The study also reported that a comparison of methane emissions across production classes, operator sizes, well status and regions produced *"almost no significant"* differences.<br><br>*Low production well site data used in this study?* **Yes**
Riddick et al. (2019) [10]	Riddick et al. measured CH_4 emissions from abandoned and active conventional wells in West Virginia using the dynamic flux chamber and Gaussian dispersion modeling approaches. The study's method focused on quantification of wellhead methane emissions. The authors reported wellhead CH_4 leakage rates for 49 low production well sites, with gas production in the range of 0.02 to 24 Mcfd and combined O&G production in the range of ~0 to 4 boed. The reported CH_4 emissions ranged from ~0 to 3.2 kg/h. The authors reported that their measured methane emission factor for active conventional wells in this state (0.138 kg/h) was a factor of 7.5 times higher than EPA's EF for these wells. Overall, they found that wellhead CH_4 emissions from active conventional wells in this state represent loss rates of 8.8% of their CH_4 production, on average.<br><br>*Low production well site data used in this study?* **Cited in the discussion, not used in the roll-up for national estimates because it focused only on wellhead emissions.**

Deighton et al. (2020)[11]	This study collected methane emissions from 43 low production well sites that produce <1 boed, focusing only on wellhead CH_4 emissions. The authors reported that the average wellhead CH_4 emission rate was 0.128 kg/h (median: 0.018 kg/h; range: 0 – 0.91 kg/h). In addition, they found that the emissions were not episodic and that some wells were emitting all or more of the gas they produced. The study did not measure emissions from tanks and the authors acknowledge that their results may be conservative. The authors attribute their observations to maintenance issues, which were prevalent at most of the sites. For example, *"some sites visited were in a state of disrepair (e.g. rusty well shafts, broken valves, fallen trees)". The authors proposed that "the main driver of emissions from the wells visited is neglect. The state of maintenance at these wells was poor. Often, pumpjacks, tanks, and other infrastructure were rusty and sometimes appeared to have temporary fixes to just keep the well mechanically operational."* The authors calculate an average CH_4 loss rate of 21%. The authors estimate that oil and gas wells in this lowest production category emit approximately 11% of total annual CH_4 from oil and gas production in the EPA greenhouse gas inventory, although they produce about 0.2% of oil and 0.4% of gas in the US per year.<br><br>*Low production well site data used in this study?* **Cited in the discussion, not used in the roll-up for national estimates because it focused only on wellhead emissions.**

Supplementary Note 5: Nonparametric Bayesian regression results

As described in the Main text, the CH_4 emissions modeling approach incorporates separate probabilistic models for the top 5% of high-emitting sites, the bottom 95% of sites and the below-detection-limit sites. For the bottom 95% of sites, CH_4 emissions are modeled using a nonparametric Bayesian regression model, defined as shown in Supplementary Fig.10. We are interested in the relationship between absolute CH_4 emissions as functions of site-level O&G production in barrels of oil equivalent per day (boed). We model the distribution (D) as a univariate normal likelihood with mean μ and standard deviation σ, where μ is a linear model with a y-intercept α and a spline basis ω, as described in the Main text.

Model implementation is achieved using the PyMC3 Bayesian modeling library.

Supplementary Fig. 10. Model definition for the nonparametric Bayesian regression model

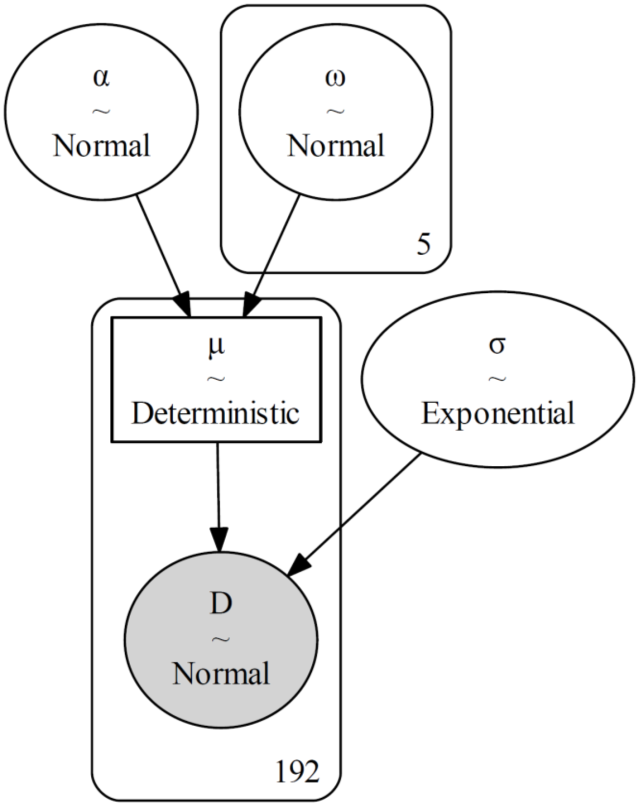

The kernel densities for the parameters α and σ are shown Supplementary Fig. 11, which also shows the plausible values from the posterior distribution. The trace plots indicate that the NUTS sampler made appropriate draws from the posterior distribution, and that good model convergence was achieved, further confirmed by the Gelman-Rubin statistic in Supplementary Fig. 12. Supplementary Fig. 13 shows the posterior predictive check for the model, indicating model predictions that closely resembles the empirical data.

Supplementary Fig. 11. Trace plots summarizing the posterior from the nonparametric Bayesian model.

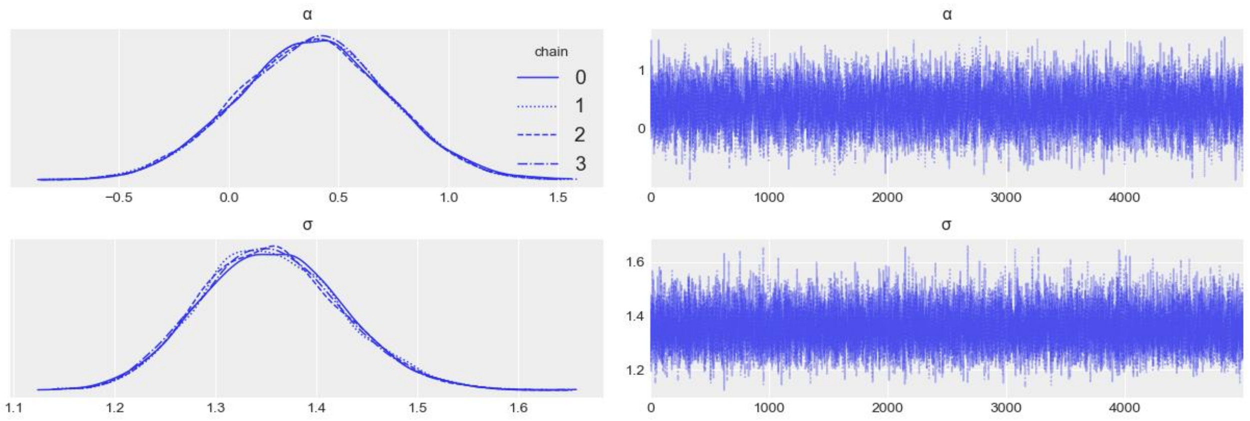

The left panel shows the Kernel Density Estimate plots, while the right panel shows the individual sampled values at each step of the Markov Chain Monte Carlo sampling using the NUTS sampler.

Supplementary Fig. 12. Forest plot showing 94% highest posterior density intervals and the Gelman-Rubin r_hat statistics for the Bayesian model.

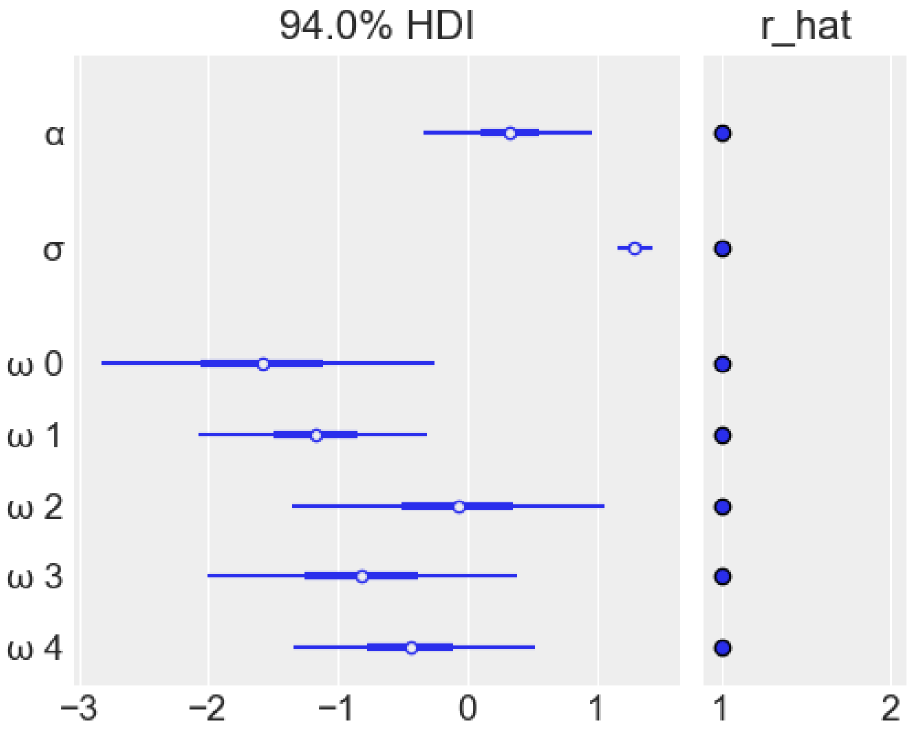

The 94% HDI means that the parameters are between the stated intervals with a probability of 94%. The Gelman-Rubic “r_hat” statistics indicate the degree of convergence of the Markov Chains.

Supplementary Fig. 13. Posterior predictive checks presented as cumulative distribution functions of the model predictions and the observed data.

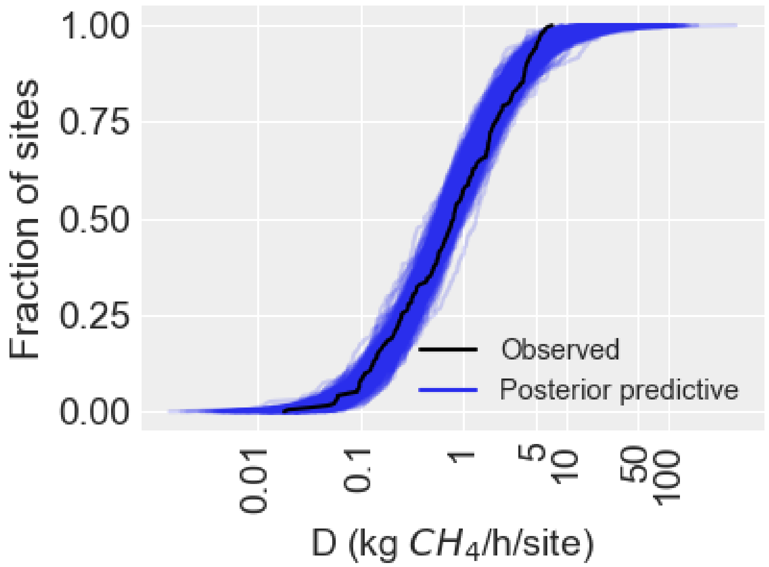

The blue lines are cumulative distribution functions of 500 samples randomly drawn from the posterior distribution. The black line shows the cumulative distribution function for the observed data.

Supplementary Fig. 14. General schematic for the emission modeling approach for low production well sites, accounting for the influence of high-emitting sites and the below-detection-limit sites (see Main Text for detailed description).

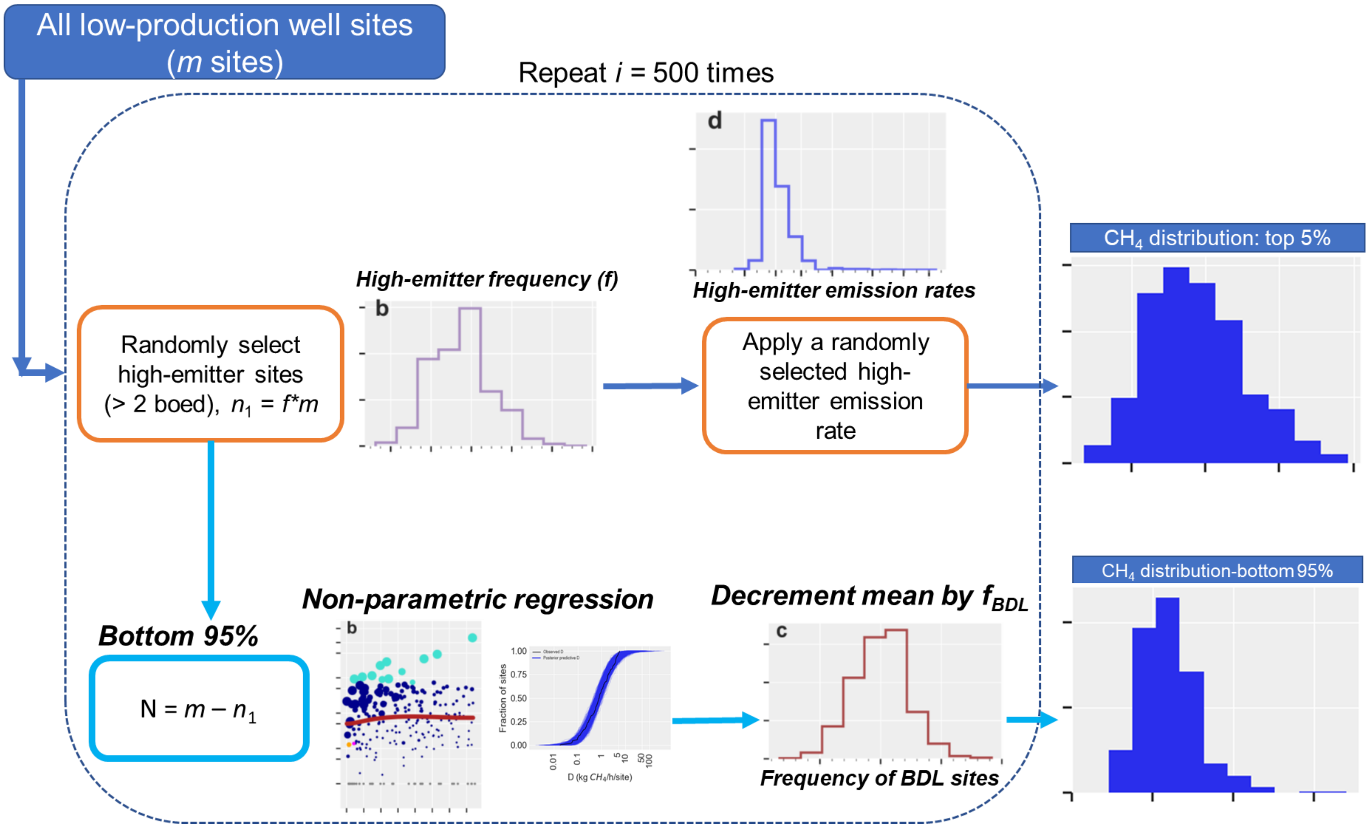

Supplementary Fig. 15. Empirical distribution of site-level methane emissions for low production well sites.

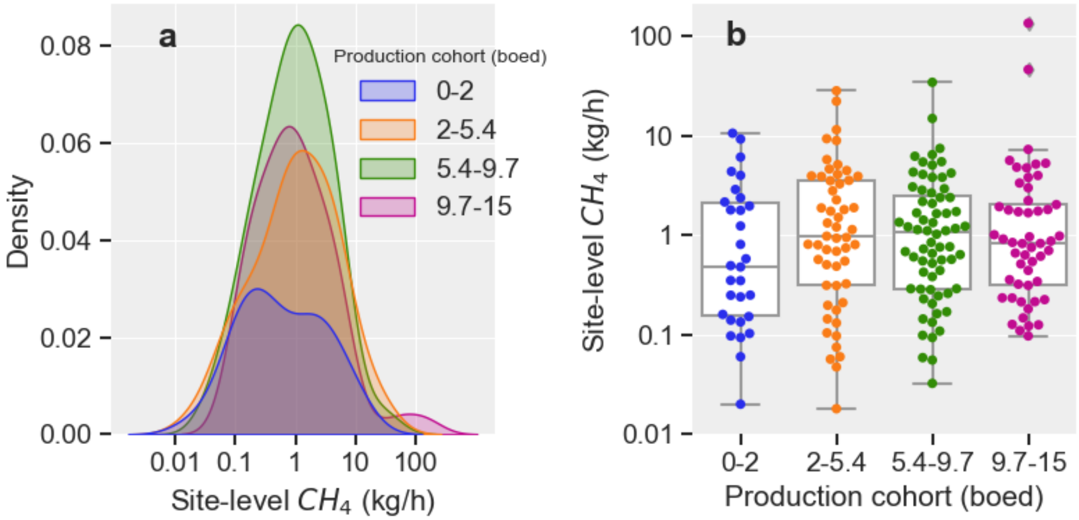

Only sites with reported methane emissions above the method detection limits are shown above.

Supplementary Note 6: An alternative model for the assessment of site-level methane emission factors

We follow the procedure in Zavala-Araiza et al.[12] and provide an alternative assessment of the site-level CH_4 emission factors for low production well sites. We split the CH_4 emission rate data into two cohorts representing the sites producing ≤2 boed (n = 41) and those producing >2 boed (n = 199). Binning the data into these two cohorts was necessary as the empirical data suggest lower CH_4 emissions for the ≤2 boed cohort (Supplementary Fig. 15). In both cases, we assume the underlying distributions are lognormal, and verify this assumption using both the Lilliefors and Shapiro-Wilk statistical tests for normality, performed on the log-transformed data for each cohort of sites. The null hypothesis for both tests is that the values are drawn from a normal distribution (unknown parameters μ and σ), with critical p-value established at 0.05.

The results of the normality tests are shown in Supplementary Table 3, indicating that one cannot reject the null hypothesis that the site-level data for both cohort of sites arise from a lognormal population distribution. We therefore use the lognormal distribution as a reasonable assumption for the site-level CH_4 data.

We fit the site-level CH_4 emissions data for each cohort of sites to a lognormal distribution, deriving the mean (μ) and standard deviation (σ) parameters using the maximum likelihood estimation approach,[12] treating data for the below-detection-limit sites (as zeros or <0.036 kg CH_4/h, n = 39) as censored data. The modeled parameters are:

(i) ≤2 boed sites: μ = -1.69 and σ = 2.399

(ii) > 2 boed sites: μ = -0.595 and σ = 2.0

The CH_4 emission factor for each cohort of sites is then computed as: $EF = e^{\mu+\frac{1}{2}\sigma^2}$. Combining these results with activity data for low production well sites results into a mean CH_4 estimate of 3.2 (95% CI: 0.8—18) kg CH_4/h/site for CH_4 emissions from low production well sites, reflecting uncertainty due to extrapolation of a small sample size to a large population of well sites. The overall emission factors are higher, but have confidence intervals that overlap with the results from our primary model, which more comprehensively assesses the distribution of emissions relative to the emitter characteristics of the high-emitting sites (top 5% of sites), the bottom 95% of sites with detectable emissions and the below-detection-limit sites.

Supplementary Fig. 16. Density plots comparing the empirical distribution of the log-transformed site-level CH_4 emission data for low production well sites producing ≤ 2 boed and > 2 *boed* sites (solid red lines) with the results of the statistical estimator based on a lognormal fit to the data (dashed blue lines).

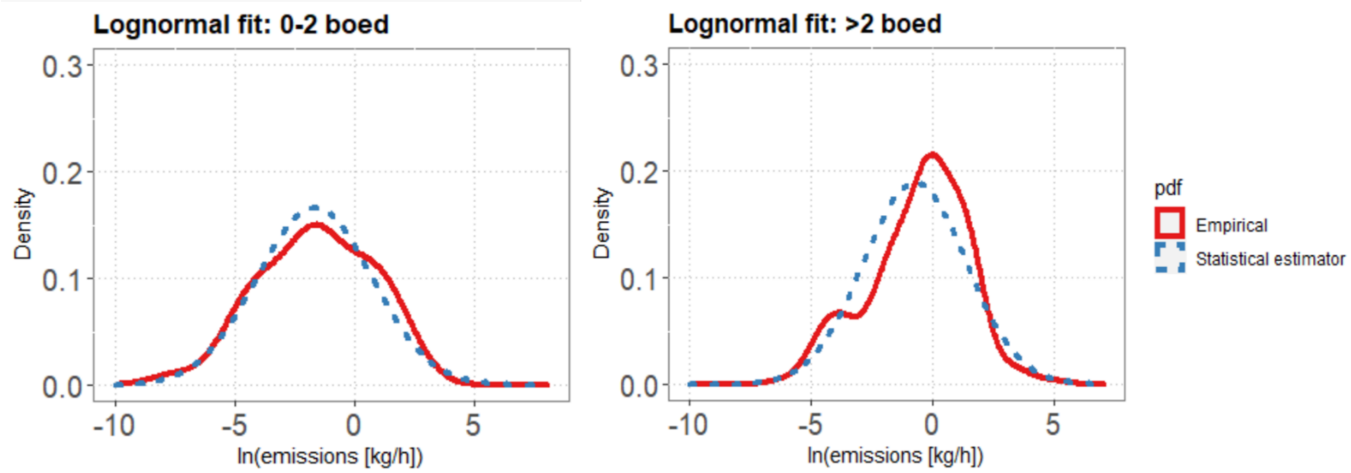

Supplementary Table 3. Results for the test for normality

	**<= 2boed**		**>2 boed**	
**Test**	***n***	***p*-value**	***n***	***p*-value**
**Lilliefors**	41	0.2553	199	0.8321
**Shapiro-Wilk**	41	0.09575	199	0.1189

Supplementary Note 7: Additional empirical evidence for extremely high methane emission rates at low production well sites.

Following similar studies in other regions[13,14] Cusworth et al.[15] performed an extensive aerial campaign using the AVIRIS-NG and GAO aircraft platforms in the Fall of 2019 to survey the Permian Basin for large CH_4 emission sources. In total they found 3,067 plumes of methane above an ~10-20 kg/hr detection limit and by use of repeated overflights aggregated their detections into 1,756 unique large emission sources by clustering all plumes within a 150m spatial buffer. Well-level production data[1] was spatially aggregated to 'well sites' as previously described (Supplementary Note 2) and spatially linked to all observations within the Cusworth et al.[15] dataset. In total, we found 78 unique plumes aggregated to 62 sources within the

Cusworth et al. dataset to emanate from well sites with 2019 annual average production less than 15 barrels-of-oil equivalent per day (boed). All 62 source locations were manually reviewed using satellite imagery to ensure the well site was isolated from emission sources on neighboring well sites or other O&G facilities by ~200m and linkable to the production data with high confidence.

Using the polygon outline of coverage area by the aircraft swath for the entire campaign, we estimate the Cusworth et al. dataset observed >95,600 well sites, with >62,600 of them meeting the ≤ 15 boed criterion to be defined as low production sites. When accounting for repeat overflights conducted on different flight days, this amounts to >194,800 observations of Permian well sites, >127,800 of which are low production. The massive extent of this campaign with respect to well site coverage suggests the rarity of observing emission plumes from low production well sites in the Permian Basin above the ~10-20 kg/hr detection limit is on the order of 0.05%, or one out of every 2,000 site visits.

The measured CH_4 emission rates in this sample are anomalously high (Supplementary Fig. 17), and may be related to site-specific safety control measures, for example, venting from pressure relief valves due to upstream overpressure conditions at tanks or separators. We note that these extreme emissions were highly impersistent: of the sites that were flown over on more than one day (37 sources), only 9 of them had more than one detection. Further studies are needed to characterize such anomalously high emission rates across a diversity of oil and gas basins in the US

Supplementary Fig. 17. Joint plot of mean site-level CH_4 emissions and their CH_4 production for 62 super-emitting low production well sites in the Permian

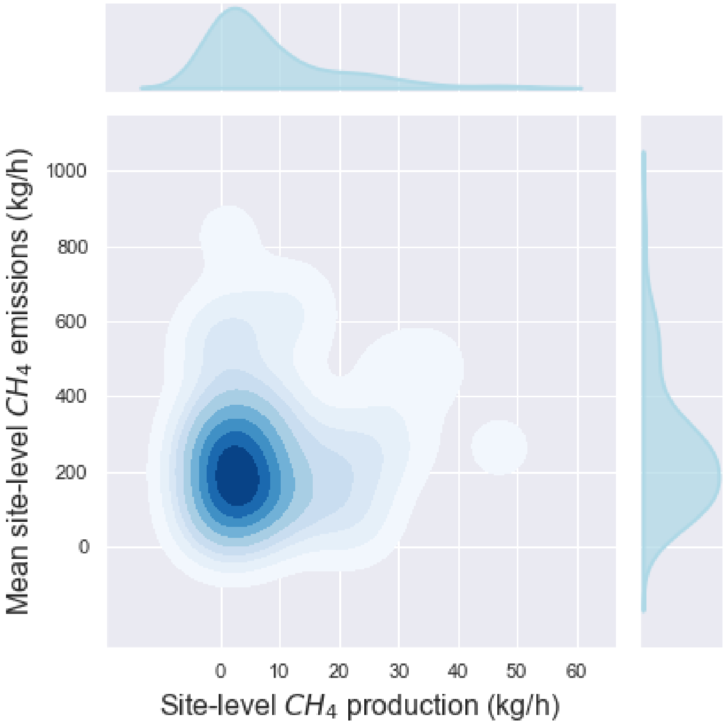

Supplementary Note 8: Representativeness of site-level data

Spatial coverage

Because the reported site-level emissions data have only limited metadata (for example, operator information and number/type of equipment on sites are often not reported), we assess sample representativeness using available data common to all datasets and specifically address (i) spatial/geographic representativeness, (ii) representativeness of production data, and (iii) representativeness of emissions data.

The 240 site-level methane emissions data are based on previous measurements conducted in seven major US oil and gas basins (Supplementary Fig. 18). These basins are diverse in their characteristics, including gas-dominant basins (e.g., Appalachian) and oil-dominant basins (e.g., Delaware and Denver-Julesburg), with average gas-to-oil ratios that range from 4 Mcf/barrel to 88 Mcf/barrel. Low production well sites in basins or regions where site-level measurement data

were collected (see Supplementary Fig. 18) account for 42% of all US low production well sites and ~20% of total US O&G production from low production well sites.

Supplementary Fig. 18. **Seven major basins for which site-level measurement-based data are available.**

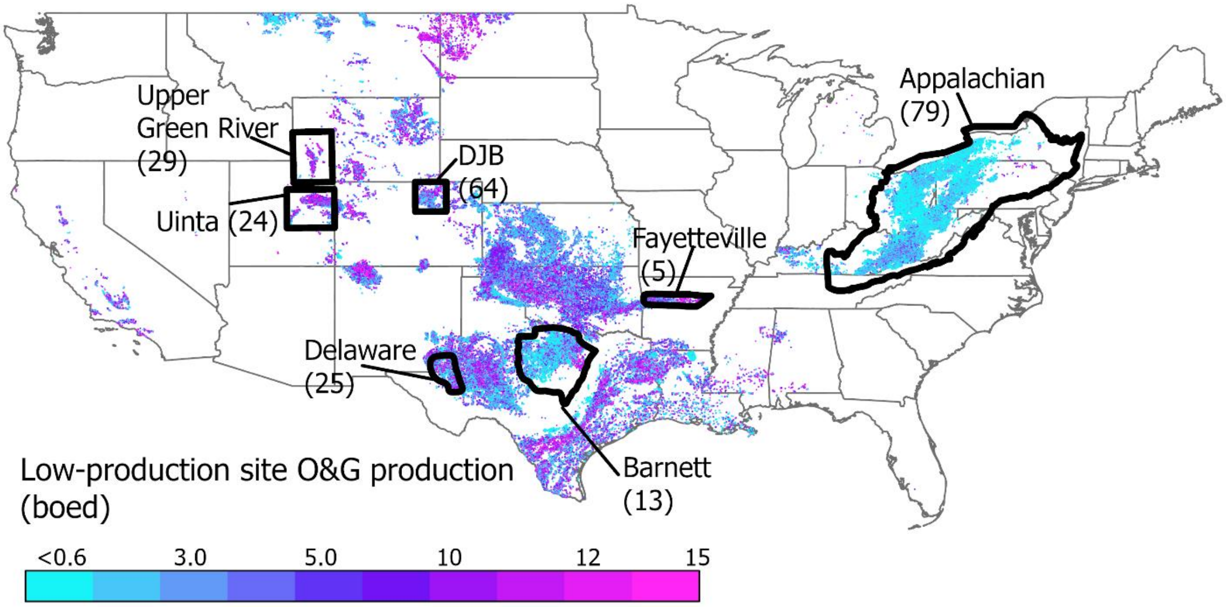

The numbers in parenthesis indicates the sample size for low production well sites extracted from respective studies. The Appalachian, Fayetteville and Delaware basin boundaries are obtained from the US EIA, while the boxes for the Upper Green River, Uinta and Denver-Julesburg are approximate basin boundaries based on descriptions as reported in the measurement-based studies.

Distribution of site-level production rates

Related to the broad spatial coverage, the production characteristics of the sampled sites are broadly representative of the production distribution for all low production well sites within the measured basins or regions and for all low production well sites nationally (Supplementary Fig. 19).

Supplementary Fig. 19. Comparison of the distribution of site-level production rates for measured sites against all low production sites in respective basins.

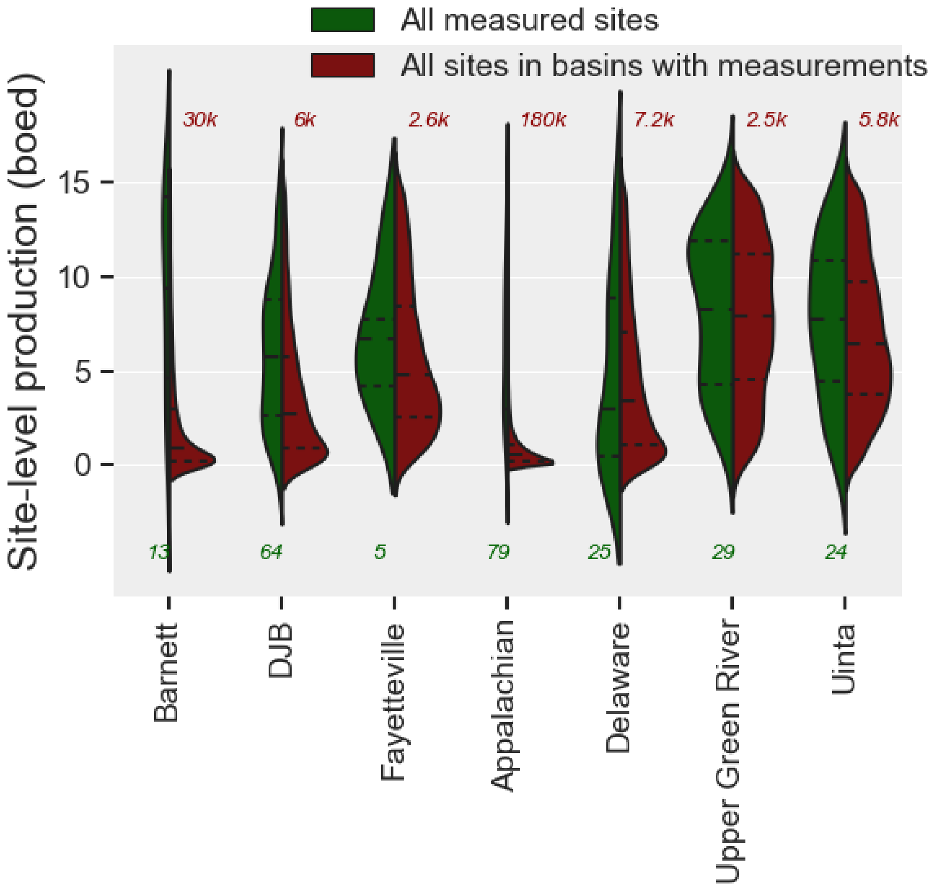

The numbers in green at the bottom of each violin shows the total sample size in the study dataset for that basin or region, while the numbers in dark red at the top of the plot (1k = 1,000) shows the total number of low production well sites in that basin or region. See Supplementary Fig. 17 for basin/region boundaries.

However, while all production cohorts (specifically, (i) < 2 boed, (ii) 2-5.4 boed, (iii) 5.4-9.7 boed and (iv) 9.7-15 boed) are represented in the measurement data, including the ultralow production cohort (<2 boed), the overall distribution for the measurements appear biased high toward low production well sites producing > ~5 boed when compared with the distribution for all sites nationally (Supplementary Fig. 19). We develop our emissions extrapolation approach

to ensure emissions characteristic of a specific production cohort are not over- or under-represented in the estimates (see Main text).

Supplementary Fig. 20. Distribution of site-level oil and gas production for all low production well sites (blue, *n* = 565,000), all low production well sites within the measured basins (orange, *n* = 236,000) shown in Supplementary Fig. 18 and all sampled sites (*n* = 218, green).

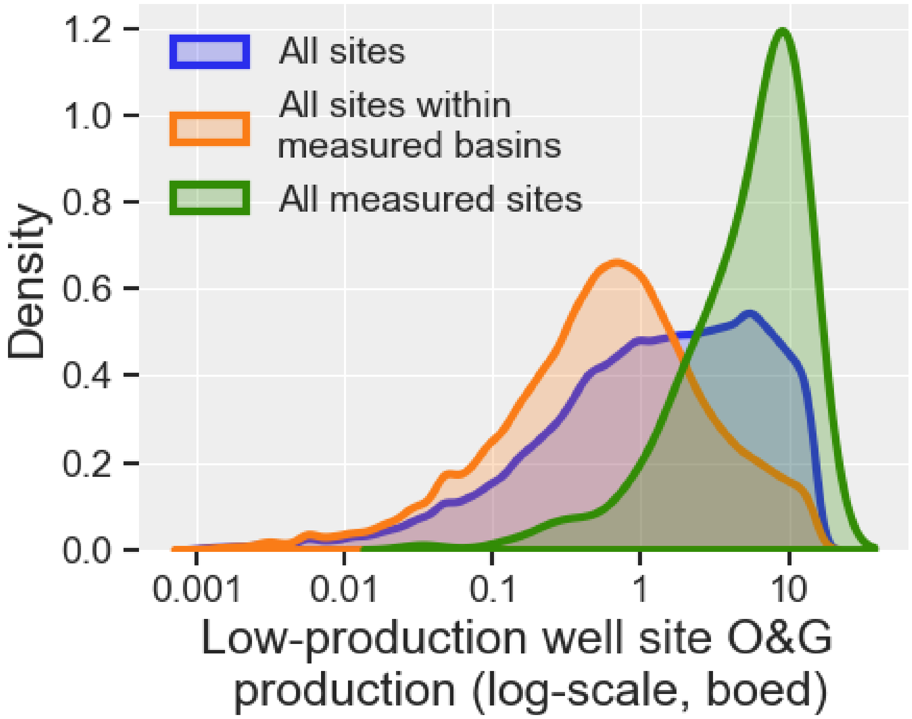

Distribution of site-level CH_4 emission rates

Because the emissions datasets are based on measurements in several basins with unique production characteristics, we assess whether the emissions distributions from specific basins are statistically similar to justify combining the datasets for purposes of estimating national-scale emissions.

Supplementary Table 4. *p*-values for the 2-sample Kolmogorov-Smirnov tests comparing site-level CH_4 emission distributions among basins.

	Appalachian	Upper Green River	Denver-Julesburg
**Appalachian**		**0.05247**	**0.0268**
**Upper Green River**	0.05247		0.0202
**Denver-Julesburg**	0.0268	0.0202	

In addition, we also simulate the stochasticity in site-level CH_4 emission distributions by randomly resampling, with replacement, 10^4 samples from the empirical distribution for these three basins. The cumulative distribution functions of the empirical and simulated distributions are shown in Supplementary Fig. 21 below, indicating considerable overlap in emission distributions among sites in these three basins.

Supplementary Fig. 21. CDF of basin-level CH_4 emissions for basins with n > 25 observations.

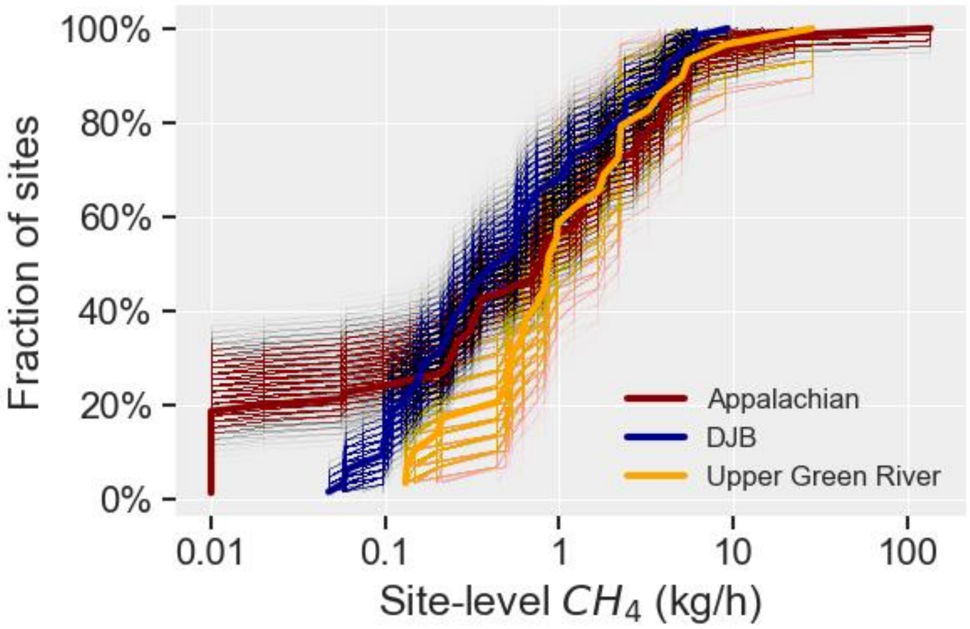

The light red, blue and orange lines show the 10^4 simulated bootstrap samples from the empirical distribution for the Appalachian, Denver-Julesburg, and Upper Green River Basins, respectively. The empirical distributions are shown in solid dark red, dark blue and dark green lines.

Supplementary Fig. 22. Modeled contribution of the top 5% of high-emitting sites to total CH_4 emissions based on empirical data

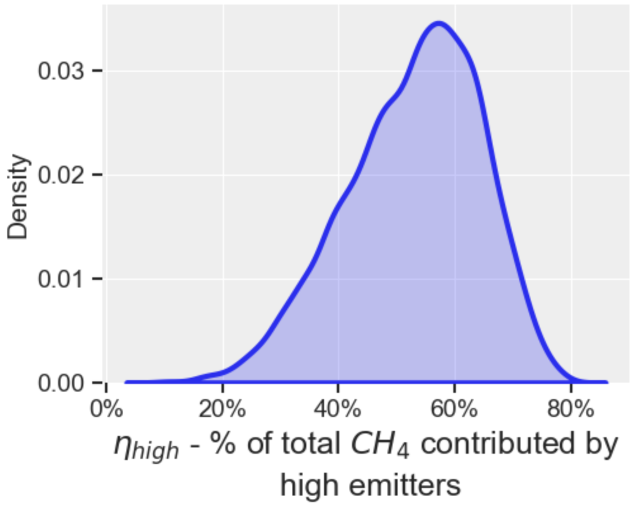

Supplementary Fig. 23. Distribution of modeled total CH_4 emissions

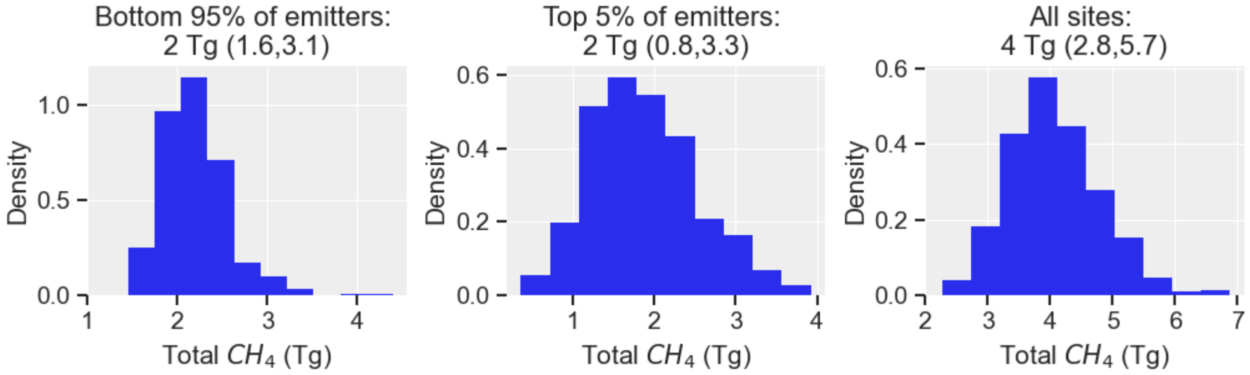

The 95% confidence intervals are shown in parenthesis and are computed based on the 2.5th and 97.th percentiles of each distribution of modeled mean methane emissions.

Supplementary Table 5. Estimated CH4 emissions for the top 5% of high-emitting sites and the remaining bottom 95% of sites.

		95% confidence interval on mean	
	**mean_ch4_emiss_Tg**	**LB_ch4_emiss_Tg**	**UB_ch4_emiss_Tg**
**Bottom 95%**	2.2	1.6	3.1
**Top 5%**	1.9	0.8	3.3
**All low production sites**	4.1	2.8	5.7
**All sites - Alvarez et al.**	7.6	6.0	9.5

Supplementary Table 6. Estimated methane emissions by production cohort

		95% confidence interval on mean				*95% confidence interval on mean*			
	mean_ch4_emiss_Tg	LB_ch4_emiss_Tg	UB_ch4_emiss_Tg	ch4_prod_Tg	mean_ch4_loss_rates_pct	LB_mean_ch4_loss_rates_pct	UB_mean_ch4_loss_rates_pct	total_number_sites	mean_ch4_per_site_kg_per_hour
**0-2 boed**	1.1	0.7	2.0	4.2	24	17	47	324,491	0.4
**2-5.4 boed**	1.5	0.9	2.2	8.2	20	11	27	124,060	1.6
**5.4-9.7 boed**	0.9	0.6	1.3	10.1	10	6	13	72,219	1.7
**9.7-15 boed**	0.6	0.3	0.8	10.5	6	3	8	44,501	1.6

Supplementary Table 7. Estimated methane emissions by study region (see Main text for region definitions)

		95% confidence interval on mean			*95% confidence interval on*	
	mean_ch4_Tg	LB_ch4_Tg	UB_ch4_Tg	mean_ch4_loss_rate_pct	LB_ch4_loss_rate_pct	UB_ch4_loss_rate_pct
**Region-1**	1.2	0.8	1.9	26	16	40
**Other**	1.0	0.7	1.3	9	7	13
**Region-4**	0.8	0.6	1.1	16	12	22
**Region-2**	0.6	0.4	0.8	7	5	10
**Region-3**	0.2	0.2	0.3	7	5	9
**Region-5**	0.2	0.2	0.3	16	12	23

Supplementary Fig. 24. Modeled emission factors for low production and non-low production sites (Rutherford et al.)

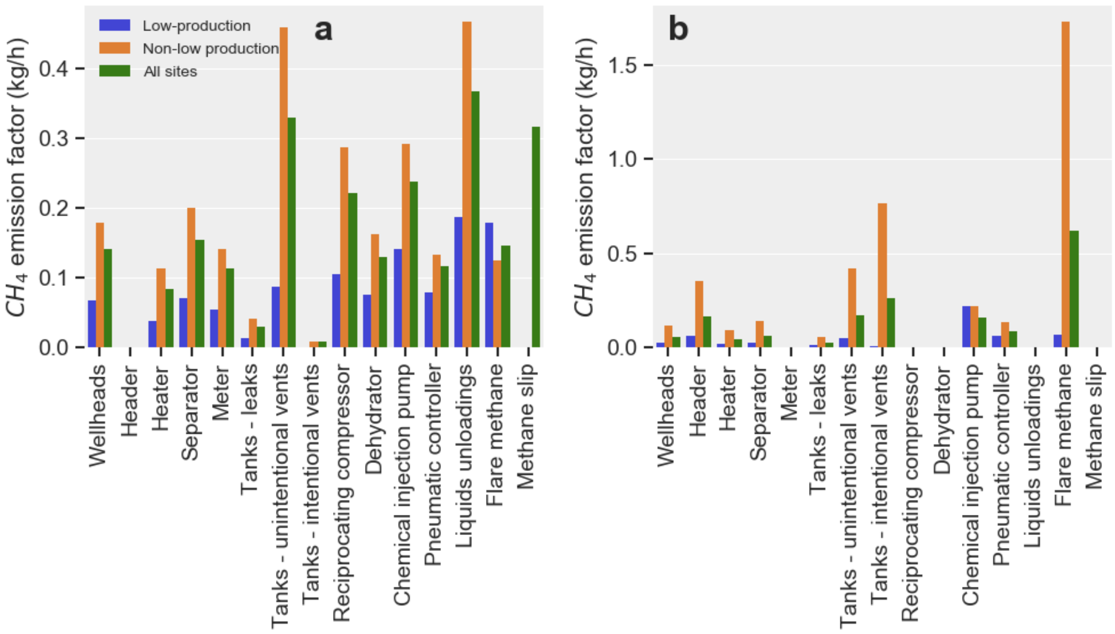

a Modeled emission factors for emission sources at natural gas well sites. B. Modeled emission factors for emission sources at petroleum systems. Note that Rutherford et al. defines low production as a site that produces <10 Mcfd of natural gas.

Supplementary Note 9: Emission estimates for operators

While our model does not directly resolve operator-specific CH_4 emissions—because the input variables are exclusive of operator information due to limited data—it is nevertheless possible to derive further insights based solely on the distribution of site count, production characteristics, and related marginal well site CH_4 emission profile. More than three-quarters of low production well sites and four-fifths of their O&G production are owned by a small fraction of operators with >100 well sites each. Treating these operators' low production well sites as a single cohort of sites and applying the same emissions modeling scheme as before, we estimate that, in aggregate, sites owned by midsize to large operators (>100 well sites each) dominate CH_4 emissions, accounting for 80% (95% CI: 62-100%) of the total.

Supplementary Table 8. An overview of US states with current O&G regulations on existing sources that may impact emissions (VOCs and/or CH_4) at low production well sites.

State	Overview of state regulations for well sites	Reference
California	O&G CH_4 regulations apply to both new and existing wells; applies to equipment leaks, pneumatic pumps, storage tanks with CH_4 > 10 metric tons per year, and compressors at well sites. Existing source regulations took effect in 2018/2019	16
Colorado	Regulations apply to both new and existing wells. Most regulations took effect in 2015, with an update for sources in the ozone non-attainment area that took effect in 2017. Includes tiered LDAR frequency at well sites tied to VOC emissions, storage tanks with VOC emissions > 6 metric tons per year, pneumatic controllers, compressors and dehydrators at well sites.	17
Utah	Applies to both new (effective 2014) and existing (effective 2018) sources. State regulations do not apply to assets on tribal lands. Regulations for well sites cover equipment leaks, tanks (with an emissions threshold), dehydrators, associated gas venting, and pneumatics.	18
Wyoming	Regulations apply to all new sources and existing sources within the Upper Green River Basin. Regulations cover equipment leaks, pneumatic controllers, tanks (with an emissions threshold), oil well completions, pneumatic pumps, and dehydrators (with an emissions threshold)	19
Texas	Texas regulations apply to new sources, relative to 2000, 2011, or 2012 depending on location and type of permit. Texas requires a leak detection and repair ("LDAR") program for certain mid-sized to large oil and gas facilities. The specific requirements vary depending on the facility's location and potential to emit uncontrolled volatile organic compounds ("VOC"). Most well sites are not subject to LDAR due to the high emissions threshold uncontrolled VOC emissions (>10 or 25 tpy) and distance from a sensitive receptor, such as a home or school, that triggers the application of LDAR.	20

Supplementary References

1. Enverus Drillinginfo (2021). https://www.enverus.com/
2. US Geological Survey, World Petroleum Assessment 2000 –Description and Results https://certmapper.cr.usgs.gov/data/PubArchives/WEcont/world/woutsum.pdf (2000).
3. US Census Bureau. https://www.census.gov/geographies/mapping-files/time-series/geo/carto-boundary-file.html (2019).

4. Omara, M., Sullivan, M., Li, X., Subramanian, R., Robinson, A.L., Presto, A.A. Methane emissions from conventional and unconventional natural gas production sites in the Marcellus Shale region. *Environ Sci Technol* **50,** 2099—2107 (2016).
5. Zavala-Araiza, D. *et al.* Toward a functional definition of methane super-emitters: application to natural gas production sites. *Environ. Sci. Technol.* **49**, 8167—8174 (2015).
6. Brantley, H.L., Thoma, E.D., Squier, W.C., Guven, B.B., Lyon, D. Assessment of methane emissions from oil and gas production pads using mobile measurements. *Environ. Sci. Technol.* **48,** 14508—14515 (2014).
7. Omara, M. *et al.* Methane emissions from natural gas production sites in the United States: data synthesis and national estimate. *Environ. Sci. Technol.* **52**, 12915-12925 (2018).
8. Robertson, A.M. *et al.* Variation in methane emission rates from well pads in four oil and gas basins with contrasting production volumes and compositions. *Environ. Sci. Technol.* **51**, 8832-8840 (2017).
9. Caulton, D. et al. Importance of superemitter natural gas well pads in the Marcellus Shale. *Environ. Sci. Technol.* **53,** 4747—4754 (2019)
10. Riddick, S. *et al.* Measuring methane emissions from abandoned and active oil and gas wells in West Virginia. *Sci. Tot. Environ.* **651**, 1849-1856 (2019).
11. Deighton, J.A., Townsend-Small, A., Sturmer, S.J., Hoschouer, J., Heldman, L. Measurements show that low production wells are a disproportionate source of methane relative to production. *J. Air Waste Manag. Assoc.* **70**, 1030-1042 (2020).
12. Zavala-Araiza, D. *et al.* Reconciling divergent estimates of oil and gas methane emissions. *Proc. Natl. Acad. Sci.* **112***,* 15597–15602 (2015).
13. Frankenberg, C. *et al.* Airborne methane remote measurements reveal heavy-tail flux distribution in Four Corners region. *Proc. Nat. Acad. Sci.* **35,** 9734-9739 (2016).
14. Duren, R. *et al.* California's methane super-emitters. *Nature* **575**, 180-184 (2019).
15. Cusworth, D.H. *et al.* Intermittency of large methane emitters in the Permian Basin. *Environ. Sci. Technol.* https://doi.org/10.1021/acs.estlett.1c00173 (2021).
16. California Code of Regulations title 17, 95665- 95677 (7- 17- 2017), available at https://govt.westlaw.com/calregs/Browse/Home/California/CaliforniaCodeofRegulations?guid=I401BB8146DA14B519A991D7827913AEE&originationContext=documenttoc&transitionType=Default&contextData=(sc.Default)
17. Colorado Air Quality Control Commission, Colorado Regulation 7, §§XII.L, effective June 30, 2018, or XVII.F, effective October 15, 2014 for well sites and January 1, 2015 for compressor stations. Available at: https://www.sos.state.co.us/CCR/DisplayRule.do?action=ruleinfo&ruleId=2341&deptID=16&agencyID=7&deptName=Department%20of%20Public%20Health%20and%20Environment&agencyName=Air%20Quality%20Control%20Commission&seriesNum=5%20CCR%201001- 9
18. Utah Administrative Code R307- 509, available at https://www.utah.gov/pmn/files/359797.pdf#page=2
19. Wyoming Department of Environmental Quality, WDEQ, Oil and Gas Production Facilities Ch. 6, Section 2 Permitting Guidance for the UGRB (2016), available at http://deq.wyoming.gov/media/attachments/Air%20Quality/New%20Source%20Revie

w/Guidance%20Documents/5-12-2016%20Oil%20and%20Gas%20Guidance.pdf. & WDEQ, Air Quality Division Rules, Chapter 8, section 6 (May 19, 2015).

20. Texas Administrative Code Title 30, Part 1, Chapter 115, Subchapter D, Division 2 and 3; available at http://texreg.sos.state.tx.us/public/readtac%24ext.ViewTAC?tac_view=5&ti=30&pt=1&ch=115&sch=D&div=2&rl=Y

21. Rutherford, J.S. *et al.* Closing the methane gap in US oil and natural gas production emissions inventory. *Nat. Comm.* **12**, 4715 (2021).
