# Supplementary material for: Methane emissions from US low production oil and natural gas well sites
Source: Nat Commun. 2022 Apr 19;13:2085. doi: 10.1038/s41467-022-29709-3 (PMC9019036; doi:10.1038/s41467-022-29709-3)
Supplement: Supplementary file 3 — Description of Additional Supplementary Files [file 41467_2022_29709_MOESM3_ESM.pdf]

Description of Additional Supplementary Files

File Name: Supplementary Data 1

Description Sitelevel methane measurement data used in the analysis.

File Name: Supplementary Data 2

Description: ESRI shapefiles for the regions
